# Assessment of the viability and mechanoresponsiveness of hMSC-TERT printed with bioinert, thermoresponsive hydrogels

**DOI:** 10.1038/s41598-025-97196-9

**Published:** 2025-04-10

**Authors:** Kirill Kriukov, Doris Schneider, Sabine Zeck, Lukas Hahn, Florian Hofmann, Stephan Altmann, Robert Luxenhofer, Regina Ebert

**Affiliations:** 1 Department of Musculoskeletal Tissue Regeneration, Orthopedic Clinic König-Ludwig Haus, University of Würzburg, Friedrich-Bergius-Ring 15, 97076 Würzburg, Germany; 2 Department of Chemistry and Helsinki Institute of Sustainability Science, Faculty of Science, University of Helsinki, PB 55, Helsinki, 00014 Finland; 3 Department for Functional Materials in Medicine and Dentistry, University of Würzburg, Pleicherwall 2, 97070 Würzburg, Germany; 4 Institute for Functional Materials and Biofabrication, University of Würzburg, Röntgenring 11, 97070 Würzburg, Germany

**Keywords:** 3D bioprinting, hMSC-TERT, Bioink rheology, Viability, Mechanoresponsiveness, Gels and hydrogels, Cell biology, Gene expression, Mesenchymal stem cells

## Abstract

During three-dimensional (3D) bioprinting, the integration of living cells into hydrogel matrices results in complex biophysicochemical interactions between viscosity, shear stress, and temperature, critically influencing the structural and functional integrity of the resulting constructs. This study delves into the short-term biological ramifications of 3D extrusion printing of telomerase-immortalized human mesenchymal stromal cells (hMSC-TERT) embedded in bioinert hydrogels. Pluronic F127 and custom-synthesized poly(2-methyl-2-oxazoline)-block-poly(2-n-propyl-2-oxazine) (POx/POzi) are synthetic, block copolymers that create shear-thinning, physically crosslinked hydrogels that were used for this study. The rheological properties of the cell-free hydrogels and cell-laden bioinks were examined, revealing that they exhibited comparable behavior. Contrary to the original hypotheses, a key finding of this research is the reduction in cell viability (up to 50%) within 24 h post-printing, a trend consistently observed across varying initial conditions. The relative expression levels of the mechanoresponsive genes FOS and PTGS2 were increased, partly due to the suspension and incubation of the cells in both hydrogels. Only FOS was significantly upregulated in some cases because of the printing process after 2 and 4 h of incubation. These insights highlight the potential of using POx/POzi hydrogel as a matrix in 3D bioprinting, particularly for depositing hMSC-TERT into structures with vasculature-mimicking scaffolds or scaffolds designed for bone regeneration.

**Supplementary Information:**

The online version contains supplementary material available at 10.1038/s41598-025-97196-9.

## Introduction

Hydrogels are natural or synthetic polymers comprising a physical or covalent 3D network that can retain substantial quantities of water^1,2^. Due to their high fluid content, biomimetic properties, and often printability, hydrogels captivated the interest of many researchers in biomaterials, drug delivery, tissue engineering, and other biomedical applications^3–6^. Various 3D bioprinting techniques enable the creation of tissue-mimicking constructs, which house cells embedded within various hydrogel matrices^7^. Hydrogels that respond to external stimuli, such as pH, light, and temperature, can be considered smart biomaterials^8^. One notable attribute of smart materials is their thermoresponsiveness, characterized by reversible phase separation above the lower critical solution temperature (LCST) or below the upper critical solution temperature (UCST)^9^. Block copolymers comprising a thermoresponsive block, such as poly(N-isopropyl acrylamide)-*block*-poly(ethylene glycol) (PNIPAM-b-PEG) or Pluronic, can form micelles above the LCST featuring a hydrophobic core and a hydrophilic outer shell^10,11^. Upon reaching the critical gelation concentration, these micelles can form thermoreversible gels due to physically entangled micellular structures^12,13^. Notably, Pluronic F127 has garnered considerable attention owing to its potential for micellar drug delivery, thermoresponsiveness, and FDA approval for pharmaceutical applications, establishing itself as the “gold standard” for thermoreversible hydrogels upon which novel hydrogels aim to improve^12,14,15^.

Another class of thermoresponsive, pseudopeptide polymers comprises closely related poly(2-oxazoline)s (POx) and poly(2-oxazine)s (POzi)^16–18^. Both polymer classes can be combined to form copolymers and block copolymers, which, depending on the multitude of side chain modifications, can change their physicochemical properties, such as changes in the LCST, gelation temperature, porosity, and printability^19–22^. Furthermore, these polymers have demonstrated high cytocompatibility and the ability to form micelles for drug delivery systems, showing promising results in vitro, in vivo, and in some clinical studies^23–25^.

While Pluronic and POx/POzi-based bioinks are widely used in biofabrication, tissue engineering, and regenerative medicine, there is a noticeable gap in systematic studies addressing the impact of extrusion printing with nonmodified, cell-laden hydrogels without chemical crosslinking on the viability and regulation of mechanoresponsive genes. Chemical crosslinking is typically employed to prevent the dissolution of otherwise soluble hydrogels, facilitating long-term incubation and comprehensive studies^26–31^.

This study used hMSC-TERT as representative models of bone marrow-derived stromal cells. Through telomerase overexpression, hMSC-TERT overcome replicative senescence, maintaining robust proliferation rates while preserving their mesenchymal differentiation capacity^32,33^. Given their versatility in multilineage differentiation, human mesenchymal stromal cells (hMSC) are essential in regenerative medicine and tissue engineering. These cells are extensively employed in additive manufacturing and are combined with diverse hydrogels and bioinks^34,35^.

Although bioinert hydrogels are generally not ideal for adherent MSC, studies suggest that prolonged incubation or encapsulation with these gels can sustain high cell viability and preserve stemness^36–38^. Given that POx/POzi is a novel hydrogel with no prior biological characterization, it was hypothesized that extended incubation could similarly support hMSC-TERT survival and differentiation. This study evaluated the viability changes through 3D printing with unmodified POx/POzi as a benchmark for future modifications, with Pluronic serving as a comparative “gold standard.” Additionally, the regulation of immediate, early, mechanoresponsive genes was investigated by utilizing the thermoresponsive nature of POx/POzi to facilitate rapid cell isolation, which would be significantly more challenging with crosslinked hydrogels. The data from this study highlight the potential of using bioinert hydrogels as biocompatible hMSC-TERT delivery systems for targeted regeneration sites^39^.

## Results and discussion

### Rheological characterization of cell-free vs. cell-laden hydrogels

Given the relatively high cell density (5 × 10^6^ cells/mL) within the bioinks utilized in this study, the impact of cells on the rheological properties of the hydrogels was assessed. According to Ouyang et al.^40^, cell densities exceeding 2 × 10^6^ cells/mL can lower the viscosity of the bioinks. To assess the changes in the rheology of the bioinks, an amplitude sweep was conducted first to determine the linear viscoelastic region (LVR). The rheological storage modulus G’ represents a material’s elastic (nonpermanent) response component to applied stress, whereas the loss modulus G’’ characterizes a plastic (permanent) component. A higher storage modulus indicates a material’s ability to store more energy.

**Fig. 1:**
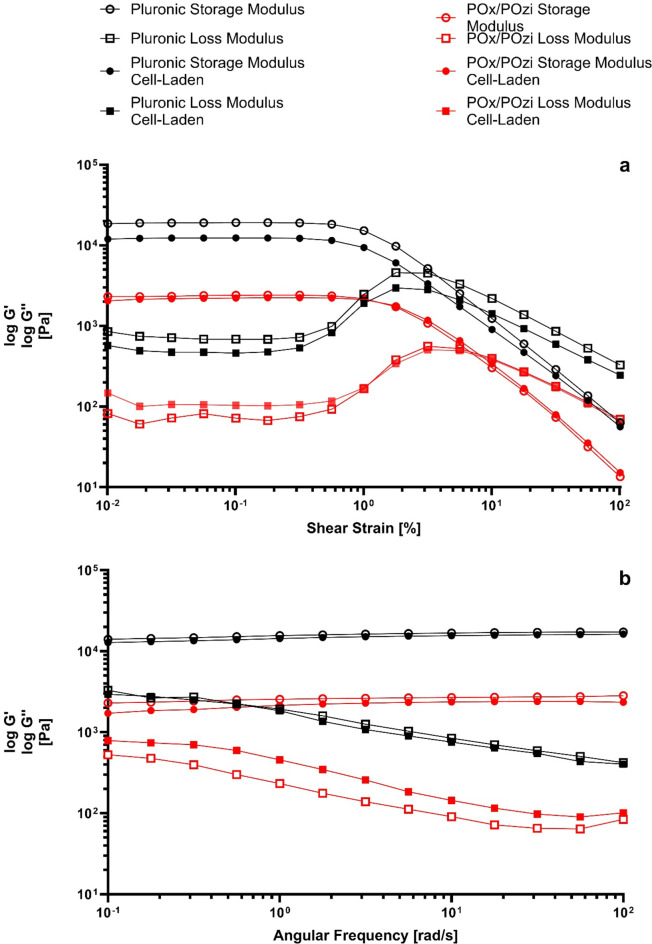
Rheological measurements of cell-free Pluronic (black), POx/POzi (red), and cell-laden bioinks at a density of 5 × 10^6^ cells/mL. All hydrogels and bioinks are at 20 w/w% polymer concentration and 37 °C. Amplitude sweeps are depicted in subfigure (**a**), whereas frequency sweeps are depicted in subfigure (**b**). The storage moduli (circles) and loss moduli (squares) represent cell-free hydrogels (empty) and cell-laden bioinks (filled), respectively.

Figure 1-a shows the storage and loss moduli of the amplitude sweep with a fixed angular frequency of 10 rad/s for both hydrogels in their cell-free and cell-laden forms. The results indicated that the LVR was approximately between 0.01 and 0.13% shear strain. From these data, a constant 0.1% shear strain was chosen for the frequency sweep measurements shown in Fig. 1-b.

The diagrams of the frequency sweep measurements show no noticeable difference in the storage and loss moduli of Pluronic and POx/POzi and their cell-free counterparts. Pluronic is a firmer gel, which is confirmed by generally higher storage moduli, loss moduli, and viscosities, as shown in the frequency sweep (Fig. 1-b) and in Supplementary Fig. 1.

Finally, the change in viscosity in relation to the shear rate in Supplementary Fig. 1 shows almost identical values between the cell-free and cell-laden gels, indicating that the rheological properties of the bioinks with a cell density of 5 × 10^6^ cells/mL are very similar to those of their cell-free hydrogel counterparts. Importantly, further increasing the cell density can lead to larger changes in the bioink properties, potentially affecting the printing capabilities of more complex structures. In conclusion, the effects of hydrogel rheology on the survival of cells during the printing process or the changes in gene expression during the post-printing process can provide valuable insight into potential applications in tissue engineering and biofabrication.

### Printing process

**Fig. 2:**
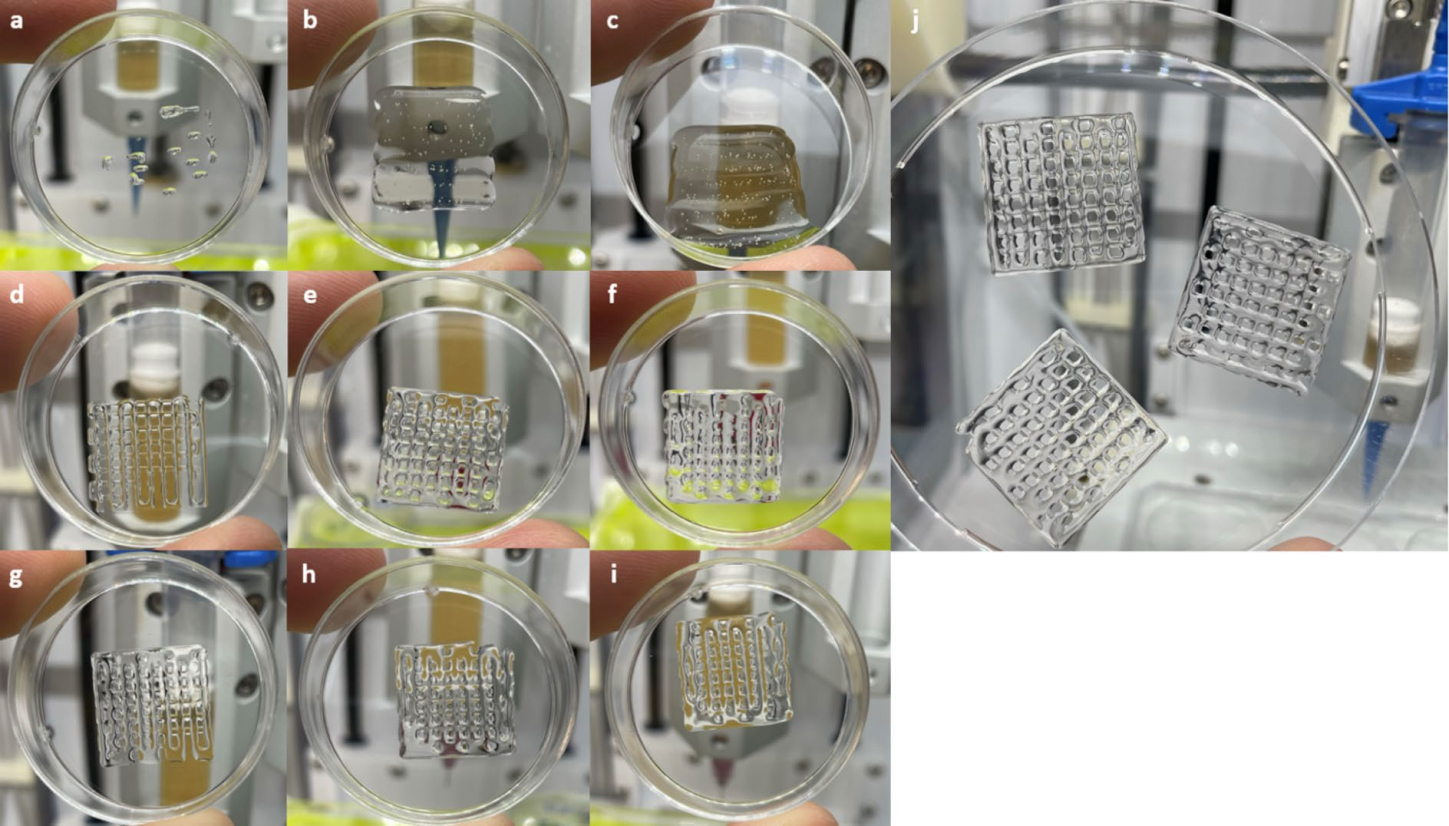
The results of POx/POzi printing pretrials showcasing varying nozzles and needle gauges. The following 16 × 16 mm grids were printed at 150 mm/min with varying printing parameters: (**a**) 22 G, 3 kPa two layers; (**b**) 22 G, 6 kPa, one layer; (**c**) 22 G, 10 kPa, one layer; (**d**) 25 G, 30 kPa, two layers; (**e**) 25 G, 35 kPa, two layers; (**f**) 25 G, 40 kPa, two layers; (**g**) 30 G, 105 kPa, two layers; (**h**) 30 G, 110 kPa, two layers; and (**i**) 30 G, 115 kPa, two layers. Subfigure (**j**) displays three 22 G, 24 × 24 mm POx/POzi grids, each composed of two layers. These grids were printed at a pressure of 7–8 kPa and a translational speed of 300 mm/min.

In the preliminary stages of this study, the printing parameters for Pluronic bioink were effectively established and optimized. However, owing to its lower viscosity (Fig. 1, Supplementary Fig. 1), further refinement was necessary for the POx/POzi bioink. 16 × 16 mm grids were printed containing at least two gel layers, initially using a 22 G nozzle, as well as 25 G and 30 G needles (Fig. 2).

While printing a 16 × 16 mm grid using a 22 G nozzle presented no difficulties with Pluronic, challenges when attempting to achieve the same grid size with POx/POzi were encountered. In these attempts, the pressure was either too low to exceed the yield stress (Fig. 2-a) or excessive material caused strand fusion (Fig. 2-b, c). To address this issue for the 22 G nozzle, the grid size was expanded to 24 × 24 mm, and the translational speed of the printing head was doubled to 300 mm/min. Additionally, a pressure of 7–8 kPa was used, which was the minimal pressure required to extrude the POx/POzi onto the printing surface. The outcomes of these alterations yielded clearly defined grid structures, as shown in Fig. 2-j. For the 30 G needle, it was determined that 110 kPa printing pressure combined with 150 mm/min translational speed (Fig. 2-h) yielded the best results within the tested set of parameters.

The morphology of the cells was characterized and compared immediately after 3D printing and after 24 h of incubation. Representative results are shown in Supplementary Fig. 2. tdTomato hMSC-TERT reporter cells were used to enhance the visibility of cell morphology. The results indicate that a portion of hMSC-TERT cells became elongated during the extrusion process. However, after 24 h, no elongated cells were observed, suggesting that the hMSC-TERT cells contracted and remained round. The cells were unable to adhere to the gel or regain their natural spindle-shaped form, remaining in this state until further analysis.

The optimal height for POx/POzi grids consists of two layers. While 10-layered grids using 20 w/w% Pluronic were successfully printed, employing the same concentration of POx/POzi without further modifications resulted in strand fusion. Although the printability of POx/POzi can be improved by adding silicate nanoparticles, such as Laponite XLG^41^, or blending with other hydrogels, such as alginate^42^, all additives were excluded to avoid potential biological effects on the printed bioinks. Nonetheless, a strategic approach to comprehensively assess the biological properties of both bioinks was adopted. Specifically, three or more separate print runs were conducted for each test group, and subsequently, the cells were pooled together after dissolution in cold PBS.

### Viability of hMSC-TERT after 3D printing

**Fig. 3:**
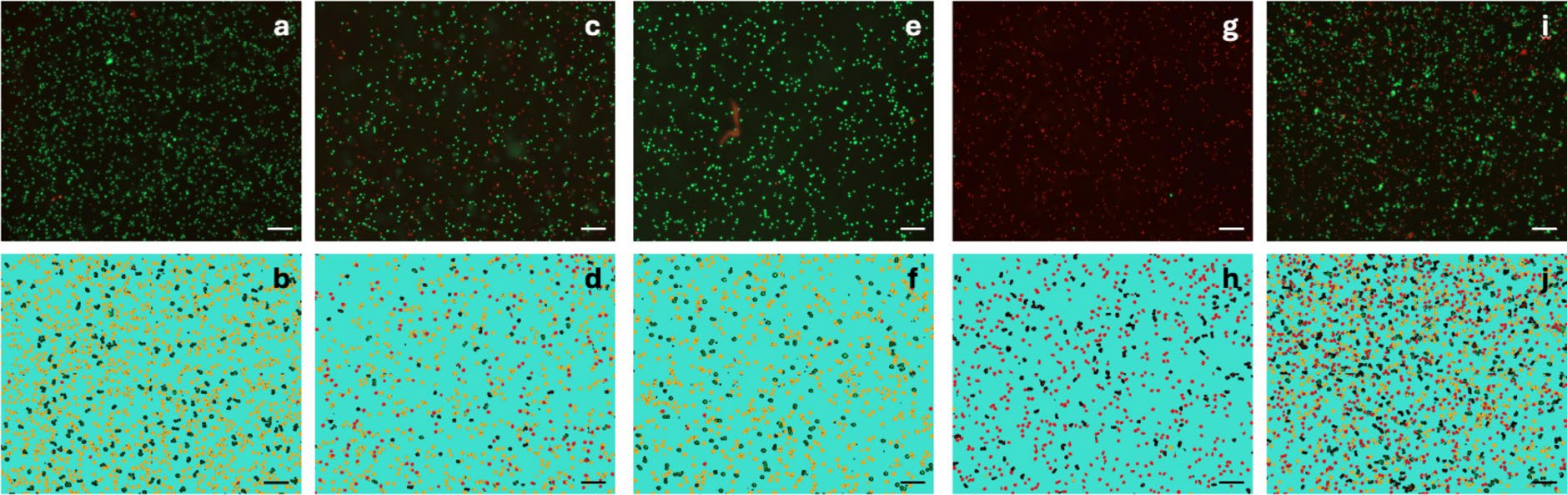
Representative live/dead imaging results as described in the “Viability assay” section of the “Materials and methods”. Subfigures (**a**), (**c**), (**e**), (**g**), and (**i**) display fluorescence images of cells stained with calcein AM (live, green) and propidium iodide (dead, red). The corresponding segmentation images generated via the ZEN Intellesis module (ZEN 2.6 Pro software) are shown in subfigures (**b**), (**d**, (**f**, (**h**), and (**j**), where the segmentation colors indicate cyan for the background, orange for live cells, red for dead cells, and transparent for omitted segments. Omitted segments were excluded based on shape and size to remove agglomerated cell clusters from viability calculations. The representative conditions shown include “Split” control (**a**, **b**), Pluronic 30 G 30 min (**c**, **d**), POx/POzi 30 G 30 min (**e**, **f**), Pluronic 30 G 24 h (**g**, **h**), and POx/POzi 30 G 24 h (**i**, **j**). The scale bars represent 200 μm. The brightness of the original fluorescence images was increased by 40% for improved visualization.

The viability assessments for the printed constructs were conducted at four distinct time points to gain insights into cell behavior over time. The “Split” control was measured from the hMSC-TERT that were isolated from the cell culture flask and were not mixed with any of the hydrogels. In the initial group (30 min), a portion of the cells was isolated immediately after printing and stained according to the protocol. Simultaneously, the remaining portions of each batch were printed into separate Petri dishes and incubated for 2 h, 4 h, or 24 h in the medium-free humidity chambers described in the “Materials and methods”. In addition, approximately 200 µL of the bioink was incubated without printing as a nonprinted “Mix” control for each corresponding time point. Figure 3 shows representative images from the live/dead evaluation, including standard fluorescence staining images and the software’s corresponding segmentation results.

**Fig. 4:**
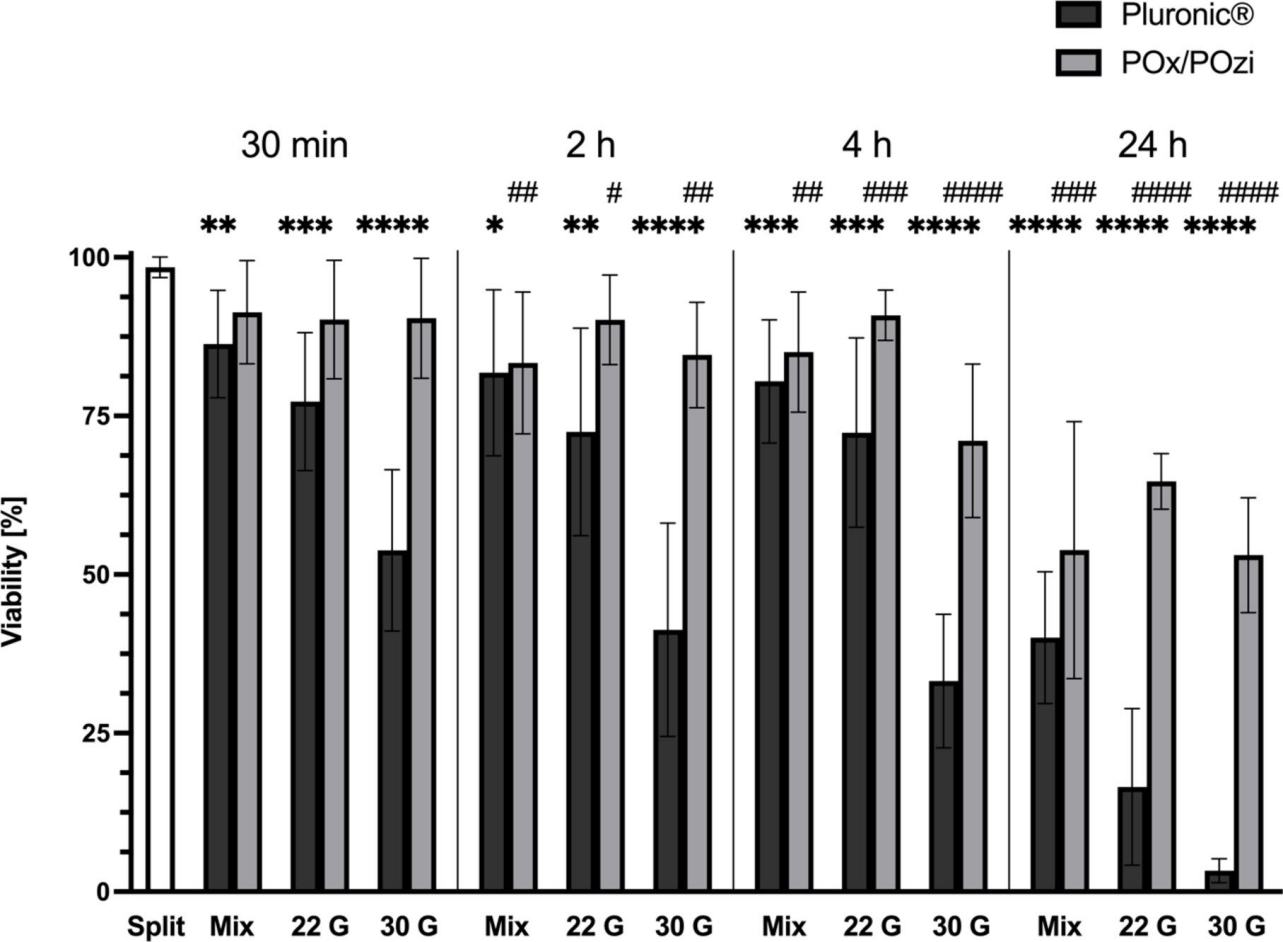
Viability test results for Pluronic (black) and POx/POzi (gray). The bar graph represents the means and standard deviations from four independent experiments, with twelve replicate measurements per data point. The “Split” control was assessed by staining the cells after they were detached from the cell culture flask. The “Mix” controls refer to the portions of bioinks that were not printed but were incubated for the same duration as the printed constructs. Statistical analysis: Brown-Forsythe and Welch ANOVA tests with Dunnett’s T3 multiple comparisons test. The “Split” control was used for Dunnets’s T3 multiple comparisons test in the statistical analysis to assess the reduction in viability relative to the ideal state. Asterisks (Pluronic) and octothorpes (POx/POzi) in the graph indicate the significance level compared with the “Split 30 min” control: */# *P*≤0.05; **/## *P*≤0.01; ***/### *P*≤0.001; ****/#### *P*≤0.0001. The results of the normal distribution tests, along with the exact P values, are provided in Supplementary Data 1.

Upon initial mixing of the hydrogels with hMSC-TERT, a reduction in cell viability to approximately 90% was observed. Printing with Pluronic using a 22 G nozzle and a 30 G needle further decreased viability to approximately 80% and 55%, respectively (Fig. 4, black bars). Interestingly, printing hMSC-TERT in POx/POzi appeared to have a relatively minor effect on cell viability, regardless of the gauge (Fig. 4, gray bars). The trend in viability within the Pluronic groups remained consistent, with a steady decline as the incubation time increased. Notably, apart from the POx/POzi “30 G 4 h” group, the viability of cells printed with this bioink remained relatively stable over the initial 4 h (> 85%).

A general observation in Fig. 4 was that cell viability declined by approximately 40–50% after 24 h of incubation, regardless of the initial printing conditions. A comparison of unprinted “Mix” controls for Pluronic between the 30-min and 24-h time points revealed a decline in viability from 86 ± 8.1% to 40 ± 9.3%. Similarly, for POx/POzi “Mix 30 min” vs. “Mix 24 h”, this viability reduction was approximately 37 ± 21%. Remarkably, the 30 G groups, particularly in the case of Pluronic, which initially displayed the lowest viability post-printing, experienced a similar decrease. For Pluronic, the viability declined between the 30 G groups by approximately 51 ± 12%, whereas for POx/POzi, this change was approximately 37 ± 13% Figs. 4-g and i and 4). The mean decline in the viability of hMSC-TERT in POx/POzi was approximately 10% lower than that in Pluronic, indicating greater cytocompatibility, which was still strongly reduced due to the lack of readherence by these cells.

The results of this study suggest that the major contributor to cell mortality is not the cytotoxicity of the gels but rather factors such as malnutrition due to a lack of excess cell medium and anoikis^43,44^, a form of apoptosis resulting from the lack of native cell-matrix contact^45^. After 4 h of incubation, both “Mix” control groups displayed viabilities exceeding 80%, confirming that the gels are not intrinsically cytotoxic. Since both polymers were dissolved in the appropriate cell medium, it was thought that the nutrients available in the hydrogels may be sufficient to support the survival of healthy cells for up to several days. However, anoikis likely contributed to the reduction in viability to a greater extent than the lack of submersion in the medium and the resulting nutrient deficiency.

The higher viability of the POx/POzi samples could be attributed to milder and more suitable printing parameters. However, this does not explain the lower average reduction in viability by approximately 10% from prolonged incubation alone, regardless of the initial printing parameters. Moreover, the primary limitation arises from the incubation duration and the embedding of adherent cells in a nonadherent gel lacking adherence sites (RGD sequences^43^) and the lack of submersion in the cell culture medium. Printing with suspension cells or chemically cross-linking the hydrogels to allow direct incubation in the cell medium would increase viability and support proliferation. However, the goal of this study was to compare nonmodified POx/POzi with a commercial, bioinert hydrogel by testing the limits of incubation conditions for adherent hMSC-TERT and evaluating how long these cells can survive in this nonnatural environment.

### Expression of mechanoresponsive genes in hMSC-TERT after 3D printing

Based on previous publications that focused on analyzing mechanoresponsive genes, attention was directed toward two key genes, namely FOS and PTGS2^46–48^. FOS, which encodes the transcription factor complex AP-1, is pivotal in regulating cell proliferation, differentiation, and transformation. Prostaglandin-endoperoxide synthase 2 (PTGS2), or cyclooxygenase-2 (COX-2), plays a key role in synthesizing prostaglandins involved in inflammatory processes and has been identified as a marker of osteogenic differentiation in MSC, a process influenced by fluid flow^49^. Although the primary functions of both genes are not mechanoregulatory, previous studies^46–48^ revealed a direct correlation between the mechanical stimulation of hMSC and the regulation of FOS and PTGS2.

**Fig. 5:**
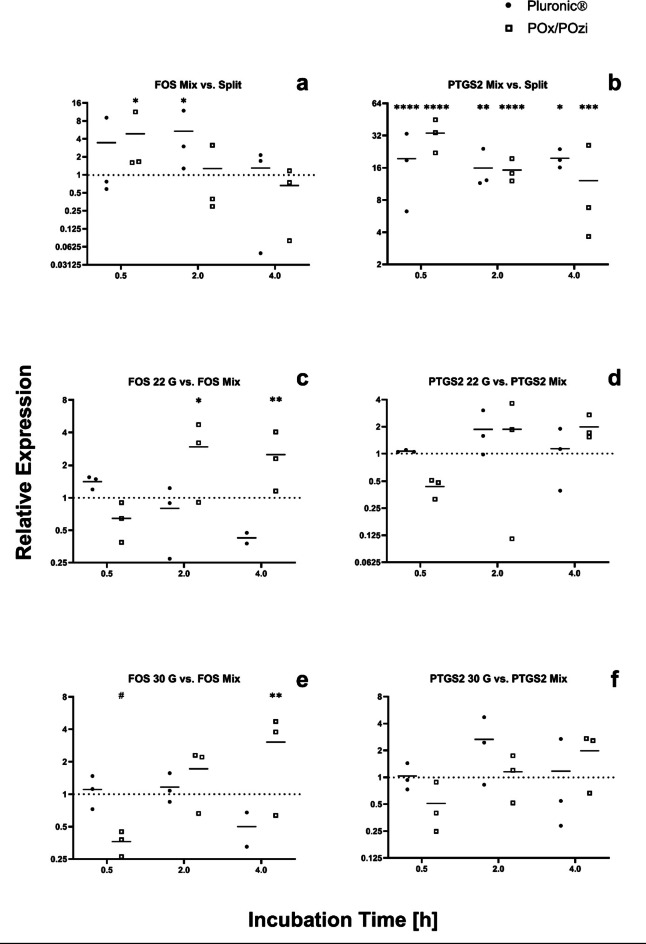
FOS and PTGS2 expression in hMSC-TERT mixed and printed with Pluronic (black dots) and POx/POzi (empty squares) hydrogels. Subfigures (**a**) and (**b**) compare the gene expression of the “Mix” controls at various time points against that of the “Split” control obtained immediately after detachment of the adherent hMSC-TERT from the flask. Subfigures (**c**)-(**f**) show the expression of FOS and PTGS2 across different time points and gauge sizes. These results were normalized to those of the corresponding “Mix” control, which was incubated for the same duration, allowing the analysis of gene regulation specifically caused by the printing process rather than time-dependent changes. The results of the qPCR measurements were evaluated via the ΔΔCt method of Pfaffl et al.^50^ and are presented as the mean values from three independent experiments, each consisting of triplicate measurements. Relative expression levels are shown on the logarithmic base-2 y-axis and were automatically generated via GraphPad Prism. The x-axis represents the incubation time in hours (not shown to scale). These changes were evaluated with REST software^51^, and significant differences between the control and test groups are indicated by asterisks (upregulation) and octothorpes (downregulation), as determined by automated statistical analysis. The significance values are as follows: */# *P*≤0.05; ** *P*≤0.01; *** *P*≤0.001; **** *P* = 0 (exact value displayed in the REST software). The exact P values are provided in Supplementary Data 2. Additional statistical analysis was conducted via an unpaired, nonparametric, two-tailed Mann-Whitney test to compare the mean expression levels within the same hydrogel at different time points. The same analysis was conducted between Pluronic and POx/POzi at the same time points. The analysis did not reveal any statistically significant differences.

To analyze and quantify the mechanical response due to 3D printing, three independent experiments with subsequent qPCR analysis of FOS, PTGS2, and ribosomal protein S27a (RPS27A) as a housekeeping gene were conducted^64^. The results are depicted in Fig. 5. The control groups are represented by the baseline at y = 1, except for Fig. 5-b, which does not display the baseline due to scaling. The ΔΔCt model published by Pfaffl et al.^50^ was utilized to analyze gene regulation. The stability of the housekeeping gene, gene regulation, and P values were assessed with the relative expression software tool (REST)^51^.

Figure 5-a and b shows the regulation of FOS and PTGS2 in the “Mix” control samples, which contained hMSC-TERT mixed with the respective hydrogels and incubated alongside the printed samples. These controls did not undergo the printing process and reflected only the temporal effects on the cells, excluding any potential influence of 3D printing. By normalizing the values to those of the “Split” control, which contained the cells recently detached from the cell culture flask via trypsinization, the gene expression of the incubated samples was compared to the optimal state of the cells before experiencing stress from suspension in the hydrogel and incubation in a nonnative environment.

The results in Fig. 5-a indicate that FOS levels increase upon mixing hMSC-TERT with both hydrogels. While the initial FOS level is regulated more strongly in POx/POzi than in Pluronic, displaying a statistically significant increase compared with the “Split” control’s baseline, it returns to the baseline by the 2-h mark. Moreover, the FOS level in Pluronic increased at the 2-h mark, indicating a statistically significant difference from the baseline and a distinct response compared with that of POx/POzi. This finding suggests that mixing the cells with either hydrogel induces a spike in FOS expression between 30 min and 2 h after cell resuspension, with the hMSC-TERT exhibiting a faster peak and return to the baseline in POx/POzi than Pluronic. These findings align with previous studies, which indicate that mechanical stimulation of MSC causes an immediate upregulation of FOS at approximately 15 min, which typically returns to baseline after 4 h^46,48^. The process of resuspending cells in the hydrogel appears to provide sufficient stimulus to induce this upregulation, which then gradually returns to the baseline in nonprinted samples.

Additionally, we analyzed the expression of PTGS2, which usually displays a similar pattern of upregulation in response to mechanical stimulus, with expression levels typically returning to baseline after 4 to 24 h^46,47^. However, Fig. 5-b shows that PTGS2 expression levels were considerably higher than FOS expression levels in Fig. 5-a and remained significantly elevated in all “Mix” control samples, regardless of the hydrogel and incubation time.

Figure 5-c-f depict the results from the printed samples normalized to the “Mix” controls. This approach ideally isolates the mechanoregulatory effects of shear stress during the printing process, excluding any influences from incubation. Figure 5-c and e show that printing cells with POx/POzi further elevated the FOS levels after 2 h (22 G nozzle) and 4 h (22 G nozzle and 30 G needle) compared with the respective 2-h and 4-h “Mix” controls. Notably, at 30 min, FOS levels either remained at the baseline (22 G sample) or were significantly downregulated in cells printed with the 30 G needle in the POx/POzi hydrogel compared with the “Mix” controls (Fig. 5-e). No effect on FOS expression could be detected in cells printed with Pluronic, regardless of the needle or nozzle. The “22 G” and “30 G” printed samples in Figs. 5-d and f did not exhibit any statistically significant changes in PTGS2 expression—either upregulation or downregulation—compared with the “Mix” samples incubated for the same duration, which showed high expression at all time points, as illustrated in Fig. 5-b.

Mechanotransduction plays a crucial role not only in influencing gene expression but also in stimulating intercellular and extracellular signaling pathways, which, in turn, leads to MSC differentiation^52^. This can be advantageous in tissue engineering, as mechanotransduction can increase the expression of cyclo-oxygenase-2 (COX-2, PTGS2) and bone morphogenetic protein 2 (BMP2) in MSC, thus promoting increased osteogenesis and differentiation toward the bone lineage^53^. However, mechanotransduction during 3D printing may induce differentiation into undesired lineages, potentially compromising the function of the printed cells.

To characterize the expression of differentiation markers in hMSC-TERT after 3D printing, the expression of the osteogenic markers ALPL (tissue non-specific alkaline phosphatase), COL1A1 (type I collagen), BGLAP (bone gamma-carboxyglutamate protein), and SPP1 (secreted phosphoprotein 1), as well as the chondrogenic markers COL2A1 (type II collagen) and ACAN (aggrecan), was analyzed (Supplementary Fig. 3). Ribosomal protein S27a (RPS27A) was used as the housekeeping gene. While the osteogenic markers ALPL and BGLAP were expressed in all samples, COL1A1 exhibited variable expression without any noticeable pattern. SPP1 was not detected in any of the samples. A similar trend was seen with the chondrogenic markers. COL2A1 was present in all samples, whereas ACAN showed no signal. Since the cells were incubated for only a short period, no significant impact on mesenchymal markers was anticipated. However, despite the reduced cell viability observed in cells printed with Pluronic, particularly when using the 30 G needle, no substantial impact on the expression of mesenchymal differentiation markers was observed.

Both FOS and PTGS2 are mechanoresponsive genes that are known to be upregulated in response to appropriate mechanical stimuli. However, since these genes also serve other functions and are influenced by various external factors, it appears that these factors outweigh the effects of shear stress from the 3D printing process. One of the key functions of the FOS gene is regulating cell proliferation, and its protein, as a component of the transcription factor AP-1, is also linked with apoptotic cell death^54^ and osteogenic differentiation. The sustained increase in FOS levels at 2 and especially 4 h may additionally be driven by extrinsic factors such as inflammation or metabolic stress, which could trigger FOS upregulation to induce apoptosis. However, this does not appear to be the case for Pluronic, which generally results in lower cell viability (Fig. 4) and higher levels of apoptosis (Supplementary Fig. 4) during 3D printing. These findings suggest that different pathways may be activated in different hydrogels despite being bioinert and are expected to have similar biological effects. The elevated and sustained PTGS2 expression may be more closely tied to inflammation-driven regulation of PTGS2 rather than the anticipated upregulation due to shear stress from the printing process.

The findings of this study indicate that the expression of mechanoresponsive genes increases significantly when the cells are mixed with the two hydrogels. Printing does not affect PTGS2 expression, regardless of the hydrogel or needle used. Similarly, FOS expression remains unchanged in Pluronic. However, in POx/POzi, the printing process significantly modulates FOS expression. These findings suggest that mixing cells with a hydrogel or incubating them in a nonnative environment has a greater effect on gene expression than extrusion and the resulting shear stress. The much stronger upregulation of PTGS2 in Fig. 5-b compared with FOS in Fig. 5-a suggests that the potential shear stress that could regulate PTGS2 expression was overshadowed mainly by inflammatory factors driving the highly significant PTGS2 upregulation in the “Mix” controls.

To minimize the undesired upregulation of these genes and the risk of differentiation into unwanted lineages, possible strategies could be to print the cells directly into the target site or to incubate them in a more native, bioactive hydrogel with adherence sites. This would allow the cells to alleviate stress and return to their baseline state. Post-printing treatments, such as chemical differentiation, can transform a printed construct into a functional scaffold with the desired lineage and biological properties. Alternatively, mechanical stimulation can increase FOS and PTGS2 expression in bone regeneration scaffolds, promoting their differentiation into the osteoblast lineage when desired^55^.

In summary, the findings of this study suggest that concerns about undesirable differentiation due to shear stress can be considered negligible in short-term applications. Additionally, an appropriate post-printing environment can help mitigate the initial adverse effects of suspending cells in hydrogels for 3D printing, ensuring better long-term viability and functionality.

## Conclusion

This study investigated POx/POzi bioink as a potential alternative to the commercially available Pluronic F127. Both hydrogels exhibit similar physicochemical properties, including cytocompatibility, bioinertness, physical crosslinking, and suitability for 3D printing. However, due to their weak physical crosslinking, immersing these hydrogels that lack chemical crosslinking into an aqueous solution causes a sol-gel transition, resulting in the dissolution of the printed structure^30,56^. This makes them unsuitable for long-term studies without further modifications. However, before such modifications can be implemented, the characterization of this novel hydrogel is essential to establish a benchmark for future improvements and applications.

The characterization focused on the short-term effects of 3D printing and subsequent incubation on the viability of hMSC-TERT and the regulation of mechanosensitive genes. The primary objective was to confirm the biocompatibility of POx/POzi and compare its printing performance to that of Pluronic. More importantly, we aimed to evaluate cellular responses to shear stress during printing, as the overexpression of mechanosensitive genes could lead to premature or unintended differentiation, potentially compromising cell functionality.

While it is generally understood that bioinert hydrogels may not be the most suitable environment for adherent MSC, research suggests that longer incubation or encapsulation with Pluronic can yield improved outcomes^36^. Studies have confirmed that MSC encapsulated in bioinert gels without adhesion sites can maintain their stemness and prolong their viability in vitro^37^ and in vivo^38^. Given their retained stemness, MSC can differentiate into different lineages without the need for adhesion sites, thereby expanding their potential applications. Printing and incubating other cell types, such as chondrocytes, might yield better viability results in bioinert hydrogels. However, chondrocytes lack the differentiation capacity of MSC, potentially limiting their range of applications. Additionally, the encapsulation of MSC or MSC-TERT may facilitate cell-cell interactions within the capsules, mimicking spheroid functionality and enabling MSC proliferation without requiring RGD sequences. However, a key prerequisite for such encapsulating hydrogels is their ability to crosslink firmly and maintain structural integrity. POx/POzi lacks this property independently and requires further modifications to achieve stable crosslinking.

Further modifications of POx/POzi are feasible, including crosslinking and adding RGD sequences into the polymer structure to enable cell adhesion^30^. Another approach for long-term studies is incorporating a more permanently crosslinkable polymer, such as alginate, into the formulation^42^. Adding nanoclays, such as Laponite XLG, might further improve the printability of the bioinks and enable the printing of more volumetric structures^57^. These methods can improve the range of applications of POx/POzi, making it a hydrogel that works as a viable scaffold in tissue engineering and regenerative medicine. However, the properties and impact of the nonmodified POx/POzi hydrogel were investigated in this study to evaluate how long cells could survive within these constructs. Characterizing the POx/POzi and assessing its biocompatibility are essential prerequisites for its potential modifications and future biological applications.

Furthermore, investigating the regulation of mechanoresponsive genes was a key focus of this study, hypothesizing that the shear stress from 3D printing would exert a more pronounced effect. A thermoresponsive, nonchemically crosslinked hydrogel facilitated easier cell isolation following the printing process. In contrast, isolating cells from a synthetic, chemically crosslinked gel would be significantly more challenging, as such gels cannot be enzymatically degraded, increasing the risk of cell damage during extraction. Furthermore, extracting cells at precise time points from organic, chemically crosslinked gels such as GelMA could pose challenges, as enzymatic degradation can also affect the cells and requires more time than thermoresponsive extraction, leading to potential measurement delays.

The qPCR results revealed that FOS was initially upregulated during the mixing process but returned to baseline levels after 4 h. In contrast, PTGS2 levels remained significantly elevated throughout the incubation period compared with those of the “Split” control, likely due to apoptosis induced by a strong inflammatory response of hMSC-TERT in a nonadherent, nonnative environment (Supplementary Fig. 4). Compared with those in “Mix” controls, the FOS levels in the printed POx/POzi 2-h and 4-h samples remained elevated, possibly because of mechanical stimulation from shear stress or other extrinsic factors, such as inflammatory and metabolic stress^54^. PTGS2 showed no significant changes when the printed samples were compared with the same controls, indicating that the stress from incubating the cells in the hydrogels outweighed the impact of the extrusion process. These results suggest that the upregulation of FOS and PTGS2 is influenced more by external factors than by shear stress, which was previously considered a major concern in 3D printing. The short bursts of shear stress either lead to complete cell death or fail to provide sufficient mechanical stimulation for meaningful and, more importantly, consistent gene regulation. Additionally, shear stress is not uniformly distributed within the printing nozzle or needle, whereas previous studies have examined MSC subjected to cyclic stimulation within their linear elastic region^48^.

While the upregulation of these mechanoresponsive genes should be considered when printing scaffolds, it may also be advantageous for applications requiring differentiation into the osteoblast lineage, such as bone regeneration^55^. Ideally, hMSC-TERT should be deposited directly into a target area, such as a healing site, or in contact with bioactive hydrogels or substrates, allowing them to reattach and regain normal functions within the 4-h period. Research suggests that Pluronic can be an effective deposition and encapsulation material for adherent MSC, supporting prolonged viability and preserving stemness under optimal conditions^39,58^. Given the similar physicochemical properties of POx/POzi, it can be a viable alternative to commercial Pluronic F127 for various applications. The findings of this study indicate greater cell viability in POx/POzi, opening possibilities for its use as a sacrificial or deposition material in biofabrication, microfluidics, and drug delivery systems^59^. Potential applications can be broadened by incorporating other cross-linkable hydrogels to facilitate interactions between diverse cell types, creating microfluidic channels that mimic the vasculature, and supporting cell attachment for improved viability and functionality^59–61^. Moreover, further research and exploration of the unique properties of POx/POzi may unveil additional innovative applications.

## Materials and methods

### Gel preparation

A commercially available Pluronic F-127 (Sigma-Aldrich, Taufkirchen, Germany) and a synthesized diblock copolymer, poly(2-methyl-2-oxazoline)-block-poly(2-*n*-propyl-2-oxazine (POx/POzi)^62^, were used as the matrix for hMSC-TERT. Lyophilized polymers (27.8 g) were dissolved by adding 100 mL of cell culture medium. The medium was composed of Eagle’s minimum essential medium (MEM) (Thermo Fisher Scientific, Heidelberg, Germany), supplemented with heat-inactivated fetal calf serum (FCS) (10 v/v%) (Bio & Sell, Ulm, Germany), gentamicin sulfate (50 µg/mL), and sodium selenite (100 nM) (both from Sigma-Aldrich). The dry polymer was autoclaved in a bottle for 20 min at 121 °C. A stirring rod was sterilized in ethanol (70 v%) and dried in a laminar flow cabinet. After the autoclaved polymer and the bottle cooled to room temperature (RT), cooled cell medium and a sterilized stirring rod were added to the bottle in the laminar flow cabinet. Finally, the bottle was closed, placed in an ice bath, and stirred overnight. The hydrogels were stored in a liquid state at 5 °C and 22 w/w% concentration.

### Cell culture and bioink preparation

hMSC-TERT cells were provided by M. Kassem, (University of Southern Denmark, Odense, Denmark)^33,63^. hMSC-TERT and hMSC-TERT tdTomato reporter cells were cultivated in the cell medium described above. The cells were grown at 37 °C and 5% CO_2_ in an environment with 95% humidified air. Bioinks with 5 × 10^6^ cells/mL were prepared. The same passage of hMSC-TERT was used for both bioinks. Initially, cells were washed with sterile phosphate-buffered saline (PBS) at 37 °C. Afterward, the cells were incubated with 1 mL of trypsin (Thermo Fisher Scientific) at 37 °C for 5 min. After incubation, the degree of cell dissociation was checked under a microscope, and the reaction was stopped with 9 mL of medium. The cell concentration was determined via a Neubauer counting chamber. The volume required for 5 × 10^6^ cells (for a 1 mL batch) was centrifuged at 230 *g* for 5 min. The supernatant was removed, and the remaining pellet was resuspended in 100 µL of cell medium. The pellet volume was not considered, and the resulting cell suspension volume was assumed to be 100 µL.

The cell suspension was placed on ice and mixed with 900 µL of gel (22 w/w%), yielding 1 mL of bioink (20 w/w%). Precooled 1000 µL Eppendorf pipette tips were used for small batches, and disposable 5 mL serological pipettes were used for larger batches. Precooling minimized bioink loss during the transfer process.

### Rheology

Rheological measurements were performed using an MCR 702 MultiDrive rheometer (Anton Paar, Ostfildern-Scharnhausen, Germany), maintaining a temperature of 37 °C and employing a parallel-plate setup with a diameter of 25 mm and a gap distance of 1 mm. The amplitude sweep test maintained a fixed angular frequency of 10 rad/s, while the shear strain ranged from 0.01 to 100%. A constant shear strain of 0.1% was employed for the frequency sweep, with frequencies ranging from 0.1 to 100 rad/s.

### Printing process

The cell-laden bioink was transferred into 3 mL Luer-Lock syringes (Cellink, Gothenburg, Sweden). Each syringe was attached to a female/female Luer-Lock adapter, which was subsequently connected to Cellink’s 3 mL printing cartridges. The gel was incubated for 10 min in an upright position in a 37 °C chamber until the bioink solidified.

A 22 G nozzle (410 μm diameter, conical) and a 30 G needle (160 μm diameter, cylindrical) (both Cellink) were selected for the 3D printing setup. The printing head (Cellink INKREDIBLE) was preheated to 37 °C. The Pluronic bioink was printed at 35 kPa using a 22 G nozzle and at 250 kPa using a 30 G needle. POx/POzi bioink was printed at 7–8 kPa using a 22 G nozzle and at 110 kPa using a 30 G needle. The POx/POzi 22 G grid comprised two 24 × 24 mm layers printed at 300 mm/min translational speed. All the other grids consisted of two 16 × 16 mm layers and were printed at 150 mm/min. Each grid consisted of two layers and nine lines per layer.

The POx/POzi 22 G grids were printed into 90 mm (diameter) polystyrene (PS) Petri dishes (Greiner Bio-One, Frickenhausen, Germany). All other grids were printed into 35 mm PS Petri dishes. During the printing process, the Petri dishes were placed on a 37 °C PCR cooler to avoid liquefaction of the printed grids. The printed constructs were then put into square 120 × 120 mm Petri dishes with a wet paper towel on the bottom and incubated at 37 °C. This construct served as a humidity chamber to prevent the gels from drying out in the medium-free environment.

To extract the cells from the bioinks, each grid was washed with 1 mL of sterile, 5 °C PBS. The cold PBS was used to liquefy the gel, thus creating a PBS-polymer solution, which was subsequently centrifuged at 230 *g* for 5 min. The cell pellet was subsequently resuspended in 1 mL of PBS, and the cells were counted for viability staining. The remaining cells were centrifuged again, and the pellet was lysed for RNA extraction.

### Viability assay

For the viability assessment, 15,000 cells were transferred into a black 96-well plate with a transparent bottom (Greiner Bio-One) and filled to 100 µL with PBS. Three replicates were selected for each printed grid. Calcein AM (1 µM) (Thermo Fisher Scientific) and propidium iodide (2 µM) (Sigma-Aldrich) were dissolved in PBS, and a mixture of both solutions was used for the viability staining. 200 µL of the staining solution was added to each well, resulting in a final volume of 300 µL per well.

The cells were incubated for 15 min at RT and analyzed with a Zeiss Axio Observer 7 fluorescence microscope (Carl Zeiss AG, Oberkochen, Germany). The analysis was facilitated by ZEN 2.6 Pro software, which included an artificial intelligence (AI) module (ZEN Intellesis) for segmenting and counting live and dead cell signals (Fig. 3). A 5x magnification was employed for the staining analysis.

Additional viability and apoptosis measurements of the printed bioink after 30 min and 24 h of incubation were assessed using Promega’s CellTiter-Glo and Caspase-Glo 3/7 assays, respectively, following the manufacturer’s protocols.

### RNA isolation, cDNA transcription, and PCR

RNA isolation was performed via the NucleoSpin RNA II kit (Machery-Nagel, Düren, Germany) according to the manufacturer’s protocol. To initiate the cDNA synthesis, the RNA (1 µg) was filled to 17 µL with nuclease-free H_2_O and annealed with Oligo(dT) primer (1 µL, 25 pmol) at 70 °C for 5 min. Subsequently, the product was reverse-transcribed with M-MLV, RNase (H-) point mutant (1 µL), reverse transcriptase (200 U/µl), 5X reaction buffer (5 µl), nuclease-free H_2_O (0.37 µL), and dNTPs (0.67 µL) (20 mM of dATP, dTTP, dGTP, dCTP each) at 42 °C for 60 min, followed by a denaturation step at 95 °C for 10 min. All reagents were purchased from Promega GmbH (Mannheim, Germany). The total volume of this product was 25 µL, and the final product was diluted with 25 µL of nuclease-free H_2_O (1:2 dilution). The cDNA product was stored at -20 °C.

For the subsequent PCR, the following reagents were used: GoTaq DNA Polymerase (0.2 µL), 5X PCR buffer (10 µL), dNTPs (0.5 µL, 20 mM), forward primer (0.5 µL, 10 pmol/µl), reverse primer (0.5 µL, 10 pmol/µl), MgCl_2_ (see Table 1), and nuclease-free H_2_O (adjusted to a total volume of 48 µL). The diluted cDNA (2 µL) was then added to the PCR mixture. All primers for the conventional PCR were purchased from Biomers GmbH, and all other reagents were purchased from Promega GmbH. The primer details are listed in Table 1.

**Table 1:** Details of the conventional PCR primers. The gene sequences, MgCl_2_ concentrations, annealing temperatures, number of cycles, and product sizes are listed below.

Primer	Primer Seq.	MgCl_2_ [µM]	Annealing Temp. [°C]	No. of Cycles	Product size [bp]
RPS27A for	TCGTGGTGGTGCTAAGAAAA	2	59	30	141
RPS27A rev	TCTCGACGAAGGCGACTAAT				
BGLAP for	ATGAGAGCCCTCACACTCCTC	2	60	40	293
BGLAP rev	GCCGTAGAAGCGCCGATAGGC				
SPP1 for	ACCCTTCCAAGTAAGTCCAA	1	58	40	352
SPP1 rev	GTGATGTCCTCGTCTGTAGC				
COL1A1 for	CCCTGGAAAGAATGGAGATG	2	58	40	150
COL1A1 rev	CCATCCAAACCACTGAAACC				
ALPL for	GTACGAGCTGAACAGGAACAACG	2	58	40	151
ALPL rev	CTTGGCTTTTCCTTCATGGTG				
ACAN for	TGGTCTTGCAGCAGTTGATTC	2	52	40	131
ACAN rev	TAGAGTCCTCAAGCCTCCTGT				
COL2A1 for	GAGCAGGAATTCGGTGTGGA	2	56	40	139
COL2A1 rev	GCCATTCAGTGCAGAGTCCT				

The PCR products were subjected to gel electrophoresis via agarose gel (1.5%) in 0.5X Tris-Borate-EDTA (TBE) buffer supplemented with GelRed (8 µL) (Biotium, Fremont, CA, USA) per 100 mL of solution as a fluorescence marker. A 100 bp DNA ladder (Low DNA Ladder 100–1.000 bp, VWR, Darmstadt, Germany) was used as a reference marker. Samples of the DNA product (15 µL) were separated at 80 V for 5 min and 100 V for 35 min.

The cDNA obtained from the synthesis above was further diluted at a 1:5 ratio, resulting in a final 1:10 dilution. The cDNA (2 µL) was mixed with GoTaq qPCR Master Mix (10 µL) (Promega GmbH), along with sequence-specific primers (0.25 pmol) sourced from biomers.net GmbH or Qiagen GmbH. The biomers primers required the addition of sense and antisense solutions (0.5 µL each) and H_2_O (7 µL). For the Qiagen primers, the primer mixture (1.5 µL) was added to H_2_O (6.5 µL). The total reaction volume for each sample was 20 µL. The following qPCR conditions were employed: an initial denaturation step at 95 °C for 3 min, followed by 40 cycles consisting of denaturation at 95 °C for 10 s, annealing at the specified temperature (refer to the Table 2) for 10 s, and extension at 72 °C for 10 s. Three independent experiments were conducted, and the qPCR experiments were carried out via the qTOWER3 thermal cycler (Analytik Jena AG, Jena, Germany). Melting curve analysis was performed to assess the specificity of the qPCR products. Differences in gene expression were calculated via the ΔΔCt method^50^, with RPS27A serving as the housekeeping gene. Gene regulation, including RPS27A, was further analyzed via the relative expression software tool (REST)^51^. The qPCR primer details are listed in Table 2.

**Table 2:** Details of the qPCR primers. The primer names, sequences, product lengths, annealing temperatures, and GenBank accession numbers are shown.

Gene Name	Primer	Primer Seq.	Annealing Temp. [°C]	Product size [bp]	Manufacturer	GenBank accession number
RPS27A^64^	RPS27A for	TCGTGGTGGTGCTAAGAAAA	60	141	Biomers GmbH	NM_00113592
RPS27A	RPS27A rev	TCTCGACGAAGGCGACTAAT				
PTGS2	Hs_PTGS2_1_SG	Qiagen Sequence	59	unknown	Qiagen	NM_000963
FOS	Hs_FOS_1_SG	Qiagen Sequence	57	unknown	Qiagen	NM_005252.3

## Electronic supplementary material

Below is the link to the electronic supplementary material.

Supplementary Material 1

Supplementary Material 2

Supplementary Material 3

## Data Availability

All raw data are available in the University of Würzburg’s repository (https://wuedata.uni-wuerzburg.de/radar/de/dataset/wqq4hjwptd1ufjze).

## References

[1] Peppas, N. A., Slaughter, B. V. & Kanzelberger, M. A. in *Polymer Science: A Comprehensive Reference* (eds Krzysztof Matyjaszewski & Martin Möller) 385–395Elsevier, (2012).

[2] Wenda, W., Ravin, N. & Hongbo, Z. in *Polymer Science and Nanotechnology* (ed Narain Ravin) 203–244Elsevier, (2020).

[3] Korah, L. V., Anilkumar, G. & Thomas, S. in *In Fundamental Biomaterials: Polymers*. 85–104 (eds Thomas, S., Balakrishnan, P. & Sreekala, M. S.) (Woodhead Publishing, 2018).

[4] Liu, S., Chen, X. & Zhang, Y. in *3D and 4D Printing of Polymer Nanocomposite Materials* (eds Kishor Kumar Sadasivuni, Kalim Deshmukh, & Mariam Alali Almaadeed) 427–465 (Elsevier, 2020).

[5] Ganguly, S., Das, P. & Das, N. C. in *In Hydrogels Based on Natural Polymers*. 481–517 (eds Chen, Y.) (Elsevier, 2020).

[6] Li, J. & Mooney, D. J. Designing hydrogels for controlled drug delivery. *Nat. Reviews Mater.* **1**, 16071. 10.1038/natrevmats.2016.71 (2016). 10.1038/natrevmats.2016.71 PMC5898614 29657852

[7] Leberfinger, A. N. et al. Bioprinting functional tissues. *Acta Biomater.* **95**, 32–49. 10.1016/j.actbio.2019.01.009 (2019). 30639351 10.1016/j.actbio.2019.01.009 PMC6625952

[8] Bordbar-Khiabani, A. & Gasik, M. Smart hydrogels for advanced drug delivery systems. *Int. J. Mol. Sci.* **23**, 3665 (2022). 35409025 10.3390/ijms23073665 PMC8998863

[9] Zhang, Q., Weber, C., Schubert, U. S. & Hoogenboom, R. Thermoresponsive polymers with lower critical solution temperature: from fundamental aspects and measuring techniques to recommended turbidimetry conditions. *Mater. Horiz.* **4**, 109–116. 10.1039/C7MH00016B (2017).

[10] Najafi, M., Habibi, M., Fokkink, R., Hennink, W. E. & Vermonden, T. LCST polymers with UCST behavior. *Soft Matter*. **17**, 2132–2141. 10.1039/d0sm01505a (2021). 33439188 10.1039/d0sm01505a

[11] Naharros-Molinero, A., Caballo-Gonzalez, M. A., de la Mata, F. J. & Garcia-Gallego, S. Direct and reverse pluronic micelles: design and characterization of promising drug delivery nanosystems. *Pharmaceutics* **14**10.3390/pharmaceutics14122628 (2022). 10.3390/pharmaceutics14122628 PMC9787366 36559122

[12] Cook, M. T., Haddow, P., Kirton, S. B. & McAuley, W. J. Polymers exhibiting lower critical solution temperatures as a route to thermoreversible gelators for healthcare. *Adv. Funct. Mater.* **31**10.1002/adfm.202008123 (2021).

[13] Shriky, B. et al. Pluronic F127 thermosensitive injectable smart hydrogels for controlled drug delivery system development. *J. Colloid Interf Sci.* **565**, 119–130. 10.1016/j.jcis.2019.12.096 (2020). 10.1016/j.jcis.2019.12.096 31945671

[14] Yu, J., Qiu, H., Yin, S., Wang, H. & Li, Y. Polymeric drug delivery system based on pluronics for cancer treatment. *Molecules* **26**, 3610 (2021). 34204668 10.3390/molecules26123610 PMC8231161

[15] Moghimi, S. M. & Hunter, A. C. Poloxamers and poloxamines in nanoparticle engineering and experimental medicine. *Trends Biotechnol.* **18**, 412–420. 10.1016/S0167-7799(00)01485-2 (2000). 10998507 10.1016/s0167-7799(00)01485-2

[16] Schlaad, H. et al. Poly(2-oxazoline)s as smart bioinspired polymers. *Macromol. Rapid Comm.* **31**, 511–525. 10.1002/marc.200900683 (2010). 10.1002/marc.200900683 21590935

[17] Varanaraja, Z., Kim, J. & Becer, C. R. Poly(2-oxazine)s: A comprehensive overview of the polymer structures, physical properties and applications. *Eur. Polym. J.* **147**10.1016/j.eurpolymj.2021.110299 (2021).

[18] Bloksma, M. M. et al. Thermoresponsive Poly(2-oxazine)s. *Macromol. Rapid Comm.* **33**, 92–96. 10.1002/marc.201100587 (2012). 10.1002/marc.201100587 22121033

[19] Lorson, T. et al. Poly(2-oxazoline)s based biomaterials: A comprehensive and critical update. *Biomaterials* **178**, 204–280. 10.1016/j.biomaterials.2018.05.022 (2018). 29945064 10.1016/j.biomaterials.2018.05.022

[20] Hahn, L. et al. Inverse thermogelation of aqueous triblock copolymer solutions into macroporous Shear-Thinning 3D printable inks. *ACS Appl. Mater. Interfaces*. **12**, 12445–12456. 10.1021/acsami.9b21282 (2020). 32142257 10.1021/acsami.9b21282

[21] Haider, M. S. et al. Tuning the thermogelation and rheology of Poly(2-Oxazoline)/Poly(2-Oxazine)s based thermosensitive hydrogels for 3D Bioprinting. *Gels* **7**, 78 (2021). 34202652 10.3390/gels7030078 PMC8293086

[22] Monnery, B. D. & Hoogenboom, R. Thermoresponsive hydrogels formed by poly(2-oxazoline) triblock copolymers. *Polym. Chem.* **10**, 3480–3487. 10.1039/C9PY00300B (2019).

[23] He, Z. et al. A high capacity polymeric micelle of Paclitaxel: implication of high dose drug therapy to safety and in vivo anti-cancer activity. *Biomaterials* **101**, 296–309. 10.1016/j.biomaterials.2016.06.002 (2016). 27315213 10.1016/j.biomaterials.2016.06.002 PMC5035646

[24] Lübtow, M. M. et al. Drug induced micellization into ultra-high capacity and stable Curcumin nanoformulations: Physico-chemical characterization and evaluation in 2D and 3D in vitro models. *J. Controlled Release*. **303**, 162–180. 10.1016/j.jconrel.2019.04.014 (2019). 10.1016/j.jconrel.2019.04.014 30981815

[25] Moreadith, R. W. et al. Clinical development of a poly(2-oxazoline) (POZ) polymer therapeutic for the treatment of Parkinson’s disease – Proof of concept of POZ as a versatile polymer platform for drug development in multiple therapeutic indications. *Eur. Polym. J.* **88**, 524–552. 10.1016/j.eurpolymj.2016.09.052 (2017).

[26] Groll, J. et al. Biofabrication: reappraising the definition of an evolving field. *Biofabrication* **8**, 013001. 10.1088/1758-5090/8/1/013001 (2016). 26744832 10.1088/1758-5090/8/1/013001

[27] Groll, J. et al. A definition of Bioinks and their distinction from biomaterial inks. *Biofabrication* **11**, 013001. 10.1088/1758-5090/aaec52 (2019). 10.1088/1758-5090/aaec52 30468151

[28] Müller, M., Becher, J., Schnabelrauch, M. & Zenobi-Wong, M. Nanostructured pluronic hydrogels as Bioinks for 3D Bioprinting. *Biofabrication* **7**, 035006. 10.1088/1758-5090/7/3/035006 (2015). 26260872 10.1088/1758-5090/7/3/035006

[29] Fedorovich, N. E. et al. Evaluation of photocrosslinked Lutrol hydrogel for tissue printing applications. *Biomacromolecules* **10**, 1689–1696. 10.1021/bm801463q (2009). 19445533 10.1021/bm801463q

[30] Hahn, L. et al. From thermogelling hydrogels toward functional Bioinks: controlled modification and cytocompatible crosslinking. *Macromol. Biosci.* **21**10.1002/mabi.202100122 (2021). 10.1002/mabi.202100122 34292657

[31] Trachsel, L., Johnbosco, C., Lang, T., Benetti, E. M. & Zenobi-Wong, M. Double-Network hydrogels including enzymatically crosslinked Poly-(2-alkyl-2-oxazoline)s for 3D Bioprinting of Cartilage-Engineering constructs. *Biomacromolecules* **20**, 4502–4511. 10.1021/acs.biomac.9b01266 (2019). 31714750 10.1021/acs.biomac.9b01266

[32] Twine, N. A. et al. Molecular phenotyping of telomerized human bone marrow skeletal stem cells reveals a genetic program of enhanced proliferation and maintenance of differentiation responses. *JBMR Plus*. **2**, 257–267. 10.1002/jbm4.10050 (2018). 30283907 10.1002/jbm4.10050 PMC6139702

[33] Abdallah, B. M. et al. Maintenance of differentiation potential of human bone marrow mesenchymal stem cells immortalized by human telomerase reverse transcriptase gene despite [corrected] extensive proliferation. *Biochem. Biophys. Res. Commun.* **326**, 527–538. 10.1016/j.bbrc.2004.11.059 (2005). 15596132 10.1016/j.bbrc.2004.11.059

[34] Zhang, J., Wehrle, E., Rubert, M. & Müller, R. 3D Bioprinting of human tissues: biofabrication, Bioinks, and bioreactors. *Int. J. Mol. Sci.* **22**10.3390/ijms22083971 (2021). 10.3390/ijms22083971 PMC8069718 33921417

[35] Ramos, T. & Moroni, L. Tissue engineering and regenerative medicine 2019: the role of Biofabrication-A year in review. *Tissue Eng. Part. C Methods*. **26**, 91–106. 10.1089/ten.TEC.2019.0344 (2020). 31856696 10.1089/ten.TEC.2019.0344

[36] Diniz, I. et al. Pluronic F-127 hydrogel as a promising scaffold for encapsulation of dental-derived mesenchymal stem cells. *J. Mater. Sci. - Mater. Med.* **26**10.1007/s10856-015-5493-4 (2015). 10.1007/s10856-015-5493-4 PMC4477746 25773231

[37] Pangjantuk, A., Kaokaen, P., Kunhorm, P., Chaicharoenaudomrung, N. & Noisa, P. 3D culture of alginate-hyaluronic acid hydrogel supports the stemness of human mesenchymal stem cells. *Sci. Rep.* **14**, 4436. 10.1038/s41598-024-54912-1 (2024). 38396088 10.1038/s41598-024-54912-1 PMC10891100

[38] Li, B. et al. Stiff hydrogel encapsulation retains mesenchymal stem cell stemness for regenerative medicine. *Matter* **7**, 3447–3468. 10.1016/j.matt.2024.05.041 (2024). 39553898 10.1016/j.matt.2024.05.041 PMC11567665

[39] Khan, S. et al. Thermoresponsive and injectable pluronic F127 hydrogel for loading Adipose-Derived mesenchymal stem cells. *Discov. Med.* **36**, 294–307. 10.24976/Discov.Med.202436181.28 (2024). 38409835 10.24976/Discov.Med.202436181.28

[40] Ouyang, L. L., Yao, R., Zhao, Y. & Sun, W. Effect of Bioink properties on printability and cell viability for 3D bioplotting of embryonic stem cells. *Biofabrication* **8**10.1088/1758-5090/8/3/035020 (2016). 10.1088/1758-5090/8/3/035020 27634915

[41] Hu, C., Haider, M. S., Hahn, L., Yang, M. & Luxenhofer, R. Development of a 3D printable and highly stretchable ternary organic-inorganic nanocomposite hydrogel. *J. Mater. Chem. B*. **9**, 4535–4545. 10.1039/d1tb00484k (2021). 34037651 10.1039/d1tb00484k

[42] Gensler, M. et al. Perfusable tissue bioprinted into a 3D-Printed tailored bioreactor system. *Bioengineering* **11**, 68 (2024). 38247945 10.3390/bioengineering11010068 PMC10813239

[43] Wang, X., Ye, K., Li, Z. H., Yan, C. & Ding, J. D. Adhesion, proliferation, and differentiation of mesenchymal stem cells on RGD nanopatterns of varied nanospacings. *Organogenesis* **9**, 280–286. 10.4161/org.26080 (2013). 23959169 10.4161/org.26080 PMC3903697

[44] Belk, L. et al. Safety considerations in 3D Bioprinting using mesenchymal stromal cells. *Front. Bioeng. Biotech.* **8**10.3389/fbioe.2020.00924 (2020). 10.3389/fbioe.2020.00924 PMC7588840 33154961

[45] Liotta, L. A. & Kohn, E. Cancer and the homeless cell. *Nature* **430**, 973–974. 10.1038/430973a (2004). 15329701 10.1038/430973a

[46] Müller-Deubert, S. et al. Phosphodiesterase 10A Is a Mediator of Osteogenic Differentiation and Mechanotransduction in Bone Marrow-Derived Mesenchymal Stromal Cells. *Stem Cells Int* (2020). 10.1155/2020/7865484 (2020). 10.1155/2020/7865484 PMC7294361 32587621

[47] Müller-Deubert, S., Seefried, L., Krug, M., Jakob, F. & Ebert, R. Epidermal growth factor as a mechanosensitizer in human bone marrow stromal cells. *Stem Cell. Res.* **24**, 69–76. 10.1016/j.scr.2017.08.012 (2017). 28843157 10.1016/j.scr.2017.08.012

[48] Ziouti, F. et al. NOTCH Signaling Is Activated through Mechanical Strain in Human Bone Marrow-Derived Mesenchymal Stromal Cells. *Stem Cells Int* (2019). 10.1155/2019/5150634 (2019). 10.1155/2019/5150634 PMC6413410 30936923

[49] Stavenschi, E., Labour, M. N. & Hoey, D. A. Oscillatory fluid flow induces the osteogenic lineage commitment of mesenchymal stem cells: the effect of shear stress magnitude, frequency, and duration. *J. Biomech.* **55**, 99–106. 10.1016/j.jbiomech.2017.02.002 (2017). 28256244 10.1016/j.jbiomech.2017.02.002

[50] Pfaffl, M. W. A new mathematical model for relative quantification in real-time RT-PCR. *Nucleic Acids Res* 29, e45, (2001). 10.1093/nar/29.9.e45 10.1093/nar/29.9.e45 PMC55695 11328886

[51] Pfaffl, M. W., Horgan, G. W. & Dempfle, L. Relative expression software tool (REST) for group-wise comparison and statistical analysis of relative expression results in real-time PCR. *Nucleic Acids Res.* **30**, e36. 10.1093/nar/30.9.e36 (2002). 11972351 10.1093/nar/30.9.e36 PMC113859

[52] Raman, N., Imran, S. A. M., Amin Noordin, A., Zaman, K. B., Nordin, F. & W. & Mechanotransduction in mesenchymal stem cells (MSCs) differentiation: A review. *Int. J. Mol. Sci.* **23**10.3390/ijms23094580 (2022). 10.3390/ijms23094580 PMC9105508 35562971

[53] Stewart, S., Darwood, A., Masouros, S., Higgins, C. & Ramasamy, A. Mechanotransduction in osteogenesis. *Bone Joint Res.* **9**, 1–14. 10.1302/2046-3758.91.Bjr-2019-0043.R2 (2020). 32435450 10.1302/2046-3758.91.BJR-2019-0043.R2 PMC7229304

[54] Preston, G. A. et al. Induction of apoptosis by c-Fos protein. *Mol. Cell. Biol.* **16**, 211–218. 10.1128/mcb.16.1.211 (1996). 8524298 10.1128/mcb.16.1.211 PMC230994

[55] Yu, H. S., Won, J. E., Jin, G. Z. & Kim, H. W. Construction of mesenchymal stem Cell–Containing collagen gel with a macrochanneled Polycaprolactone scaffold and the flow perfusion culturing for bone tissue engineering. *BioResearch Open. Access.* **1**, 124–136. 10.1089/biores.2012.0234 (2012). 23515189 10.1089/biores.2012.0234 PMC3559226

[56] Sakai, T., Katashima, T., Matsushita, T. & Chung, U. -i. Sol-gel transition behavior near critical concentration and connectivity. *Polym. J.* **48**, 629–634. 10.1038/pj.2015.124 (2016).

[57] Hu, C. et al. A thermogelling organic-inorganic hybrid hydrogel with excellent printability, shape fidelity and cytocompatibility for 3D Bioprinting. *Biofabrication* **14**, 025005. 10.1088/1758-5090/ac40ee (2022). 10.1088/1758-5090/ac40ee 34875631

[58] Vashi, A. V. et al. Adipose differentiation of bone marrow-derived mesenchymal stem cells using pluronic F-127 hydrogel in vitro. *Biomaterials* **29**, 573–579. 10.1016/j.biomaterials.2007.10.017 (2008). 17980905 10.1016/j.biomaterials.2007.10.017

[59] Kolesky, D. B. et al. 3D Bioprinting of vascularized, heterogeneous Cell-Laden tissue constructs. *Adv. Mater.* **26**, 3124–3130. 10.1002/adma.201305506 (2014). 24550124 10.1002/adma.201305506

[60] Bingchu, P. et al. 3D printing sacrificial templates for manufacturing hydrogel constructs with channel networks. *Mater. Des.* **222**10.1016/j.matdes.2022.111012 (2022).

[61] Neufeld, L. et al. Microengineered perfusable 3D-bioprinted glioblastoma model for in vivo mimicry of tumor microenvironment. *Sci. Adv.* **7**, eabi9119. 10.1126/sciadv.abi9119 (2021). 34407932 10.1126/sciadv.abi9119 PMC8373143

[62] Lorson, T. et al. A thermogelling supramolecular hydrogel with Sponge-Like morphology as a cytocompatible Bioink. *Biomacromolecules* **18**, 2161–2171. 10.1021/acs.biomac.7b00481 (2017). 28653854 10.1021/acs.biomac.7b00481

[63] Simonsen, J. L. et al. Telomerase expression extends the proliferative life-span and maintains the osteogenic potential of human bone marrow stromal cells. *Nat. Biotechnol.* **20**, 592–596. 10.1038/nbt0602-592 (2002). 12042863 10.1038/nbt0602-592

[64] Noriega, N. C., Kohama, S. G. & Urbanski, H. F. Microarray analysis of relative gene expression stability for selection of internal reference genes in the rhesus macaque brain. *Bmc Mol. Biol.* **11**10.1186/1471-2199-11-47 (2010). 10.1186/1471-2199-11-47 PMC2914640 20565976

