# Supplementary material for: Assessment of the viability and mechanoresponsiveness of hMSC-TERT printed with bioinert, thermoresponsive hydrogels
Source: Sci Rep. 2025 Apr 10;15:12257. doi: 10.1038/s41598-025-97196-9 (PMC11986050; doi:10.1038/s41598-025-97196-9)
Supplement: Supplementary file 3 — Supplementary Material 3 [file 41598_2025_97196_MOESM3_ESM.docx]

# Supplementary Information

Assessment of the Viability and Mechanoresponsiveness of hMSC-TERT Printed with Bioinert, Thermoresponsive Hydrogels

*Kirill Kriukov ^1^, Doris Schneider^1^, Sabine Zeck^1^, Lukas Hahn^4^, Florian Hofmann^3^, Stephan Altmann^1^, Robert Luxenhofer^2,4*^, Regina Ebert^1*^*

^1^ Department of Musculoskeletal Tissue Regeneration, Orthopedic Clinic König-Ludwig Haus, University of Würzburg, Friedrich-Bergius-Ring 15, 97076 Würzburg, Germany

^2^ Department of Chemistry and Helsinki Institute of Sustainability Science, Faculty of Science, University of Helsinki, PB 55, 00014, Helsinki, Finland

^3^ Department for Functional Materials in Medicine and Dentistry, University of Würzburg, Pleicherwall 2, 97070 Würzburg, Germany

^4^ Institute for Functional Materials and Biofabrication, University of Würzburg, Röntgenring 11, 97070 Würzburg, Germany

Correspondence:

regina.ebert@uni-wuerzburg.de; Tel.: +499318031597

robert.luxenhofer@helsinki.fi; Tel.: +358294159160


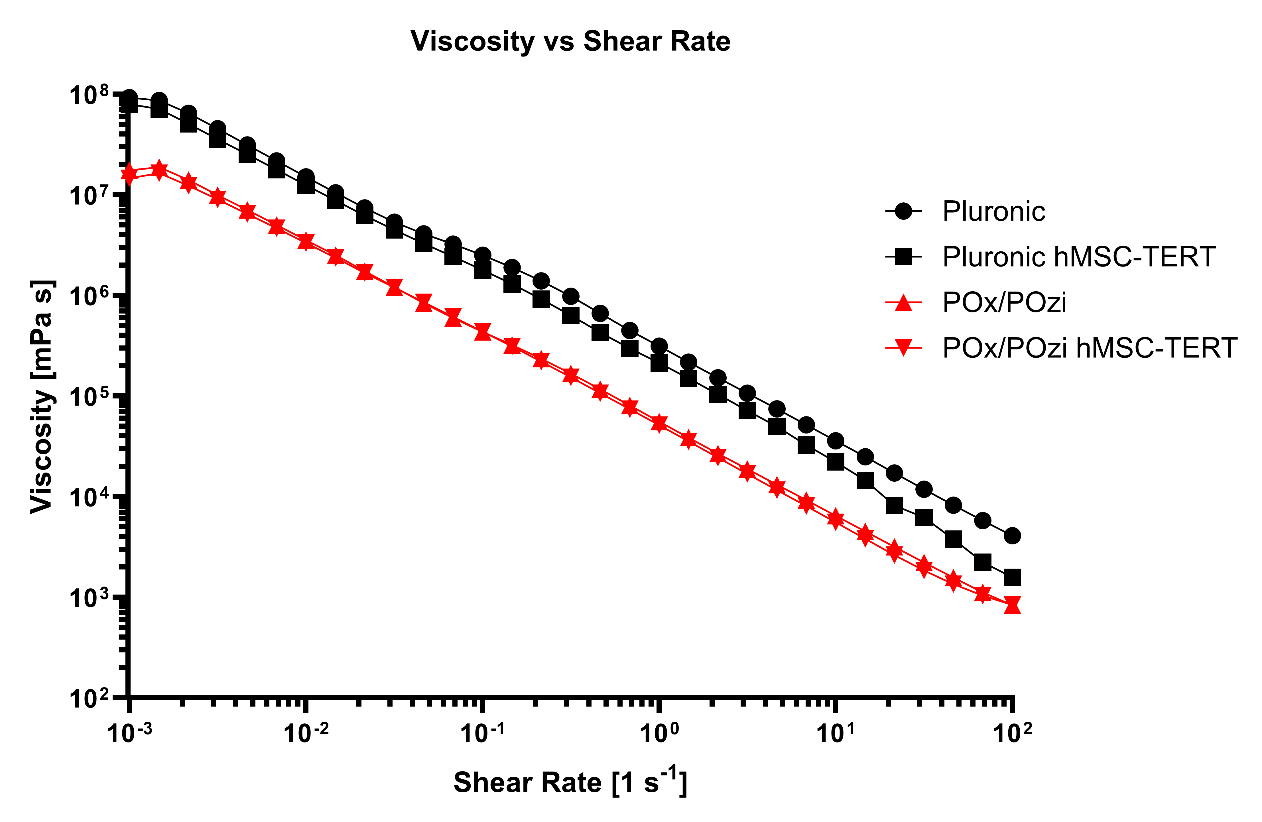


**Supplementary Figure 1.** Viscosity change plotted against increasing shear rate. Both hydrogels reach a flow point at the shear rate of approximately 10.1^-3^ s^-1^. The graph illustrates the lower viscosity of POx/POzi compared to Pluronic, with only a slight decrease in viscosity due to the addition of hMSC-TERT into the bioinks.

**
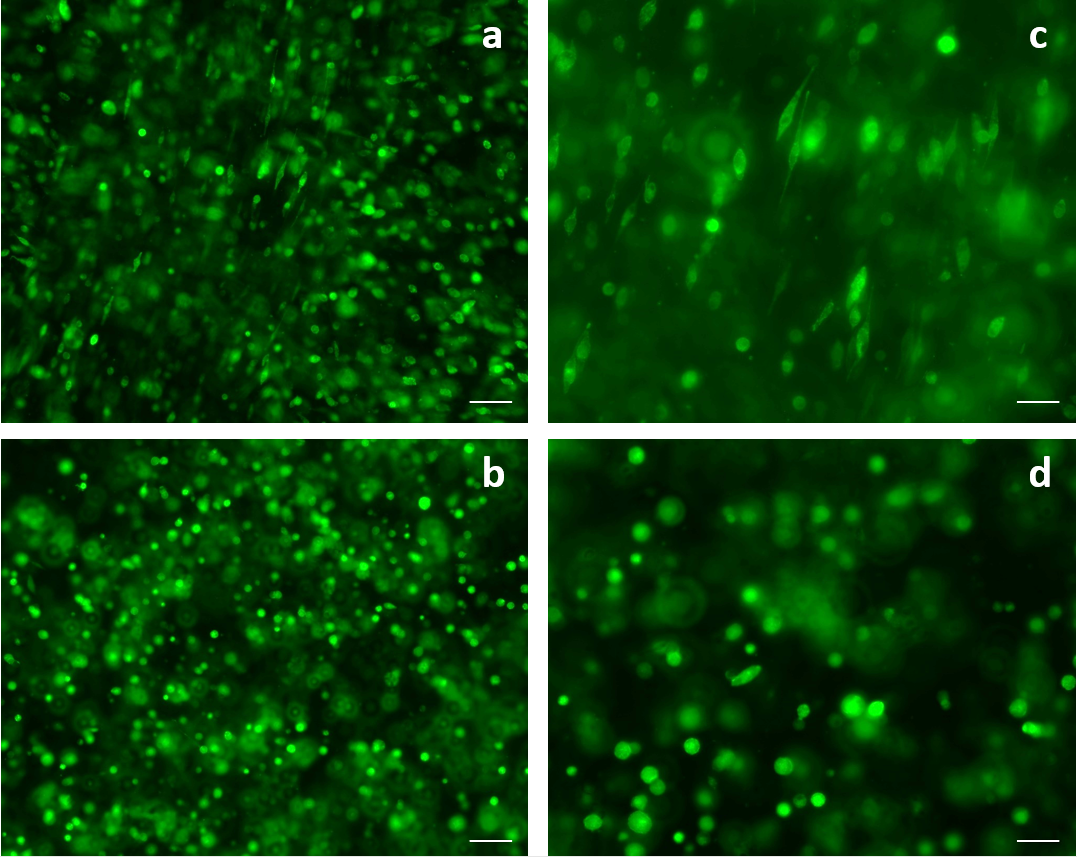
**

**Supplementary Figure 2**. Representative morphology characterization of hMSC-TERT tdTomato reporter cells printed with POx/POzi using a 22 G nozzle. The subfigures are as follows: **a** – 10× magnification, immediately after printing; **b** – 10× magnification, after 24 hours of incubation; **c** – 20× magnification, immediately after printing; **d** – 20× magnification, after 24 hours of incubation. **a** and **c** show that some cells appeared elongated immediately after printing. However, after 24 hours, no elongated cells were observed, suggesting that the cells contracted during incubation without the ability to attach to other cells or adhesion sites. The scale bars for **a** and **b** represent 100 µm, while the scale bars for **c** and **d** represent 50 µm. The green fluorescence color was artificially applied to enhance contrast, and the brightness of the original images was increased by 40% for improved visualization.

The tdTomato vector was linearized with PvuI (New England Biolabs, Frankfurt am Main, Germany) and hMSC-TERT were transfected with Lipofectamine™ 3000 (Thermo Fisher Scientific) according to the manufacturers recommendations. Stable cells were selected with 1 µg/mL puromycin (Thermo Fisher Scientific). The cells were printed as described in the Materials and Methods section, and images were captured at an excitation wavelength of 555 nm.


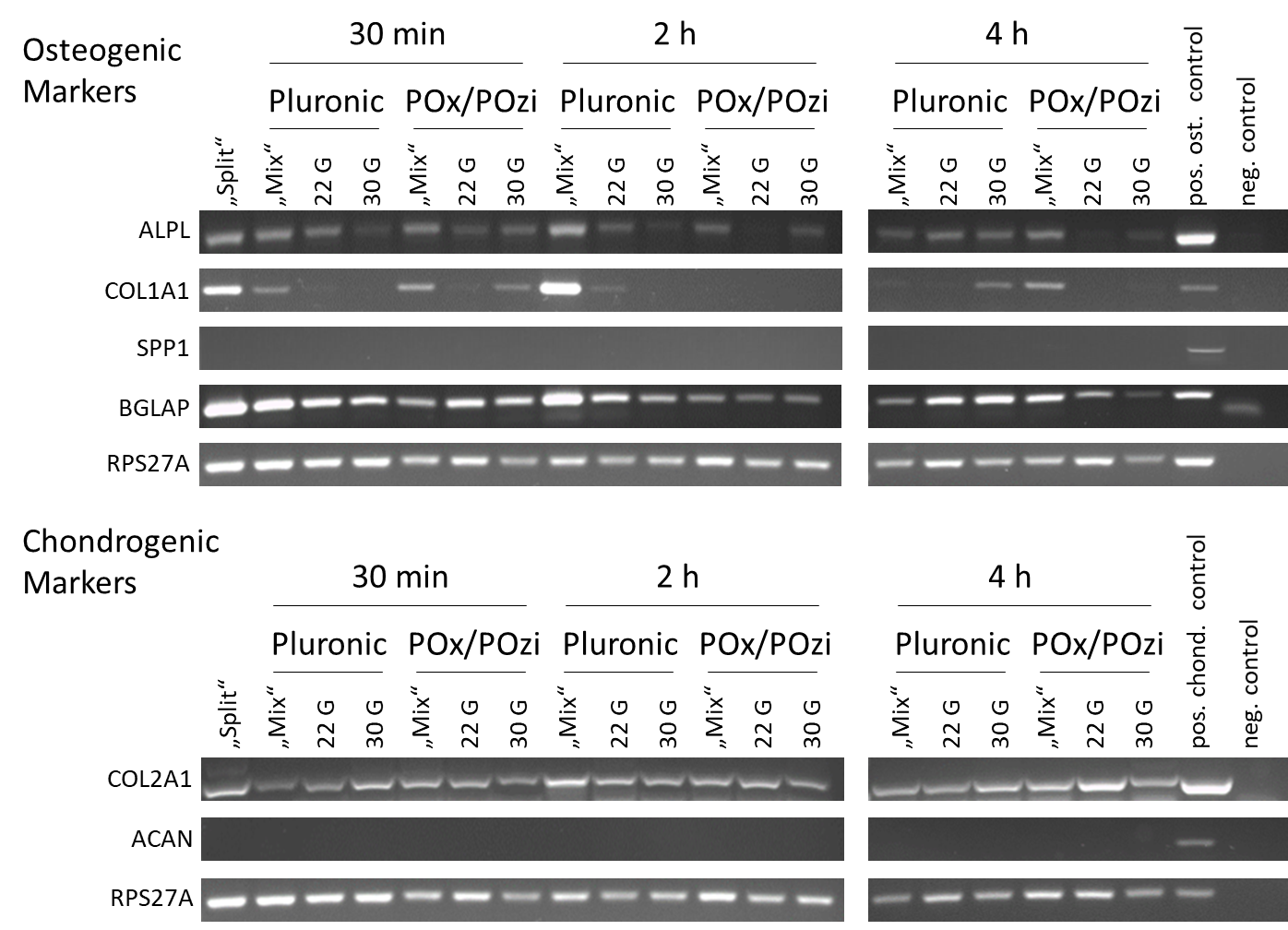


**Supplementary Figure 3.** Expression of osteogenic and chondrogenic markers after 3D printing. Gene expression of the “Split” control, the “Mix” controls and of hMSC-TERT printed with Pluronic and POx/POzi by using the 22 G nozzle or the 30 G needle after 30 min, 2 h, and 4 h are shown. Osteogenic markers: ALPL (tissue non-specific alkaline phosphatase), COL1A1 (type I collagen), BGLAP (bone gamma-carboxyglutamate protein), SPP1 (secreted phosphoprotein 1); chondrogenic markers: COL2A1 (type II collagen) and ACAN (aggrecan) were analyzed. Ribosomal protein S27a (RPS27A) was used as housekeeping gene. Three independent experiments were performed; uncropped images are depicted below.

Uncropped images


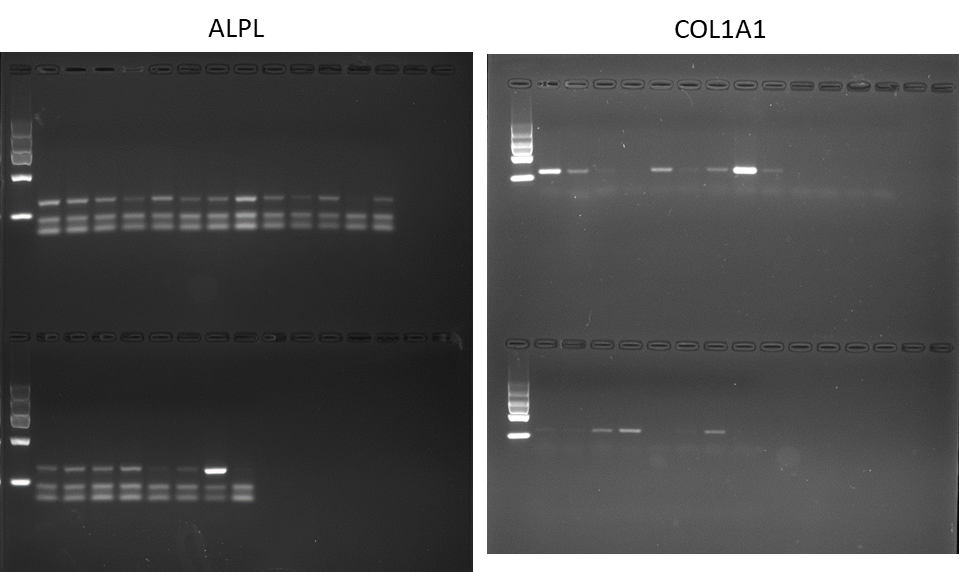


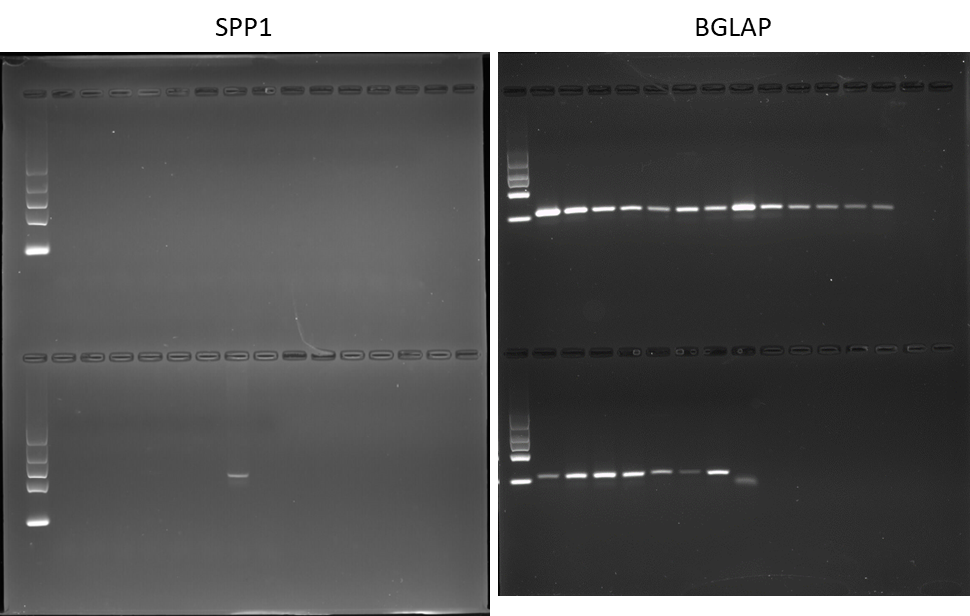


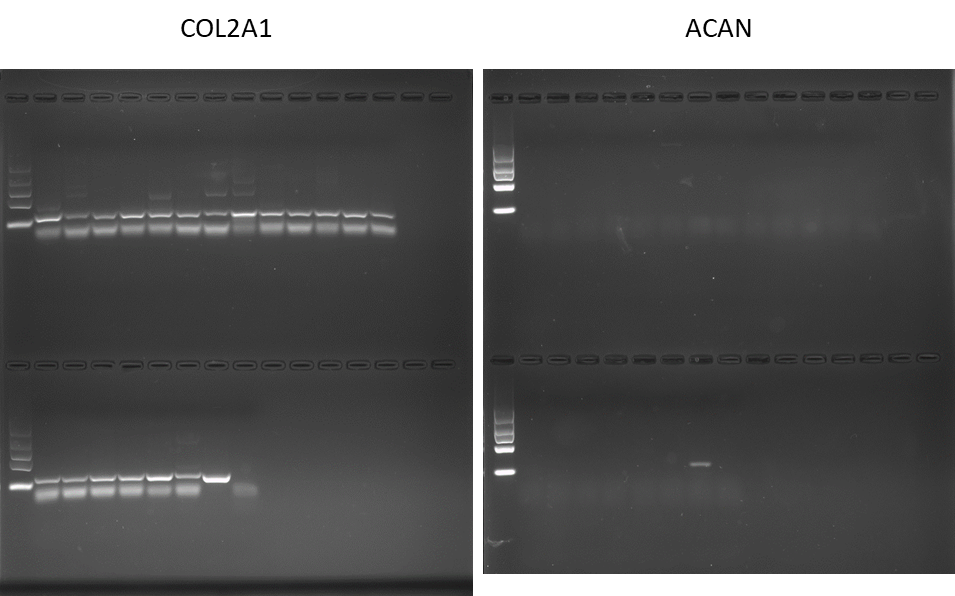


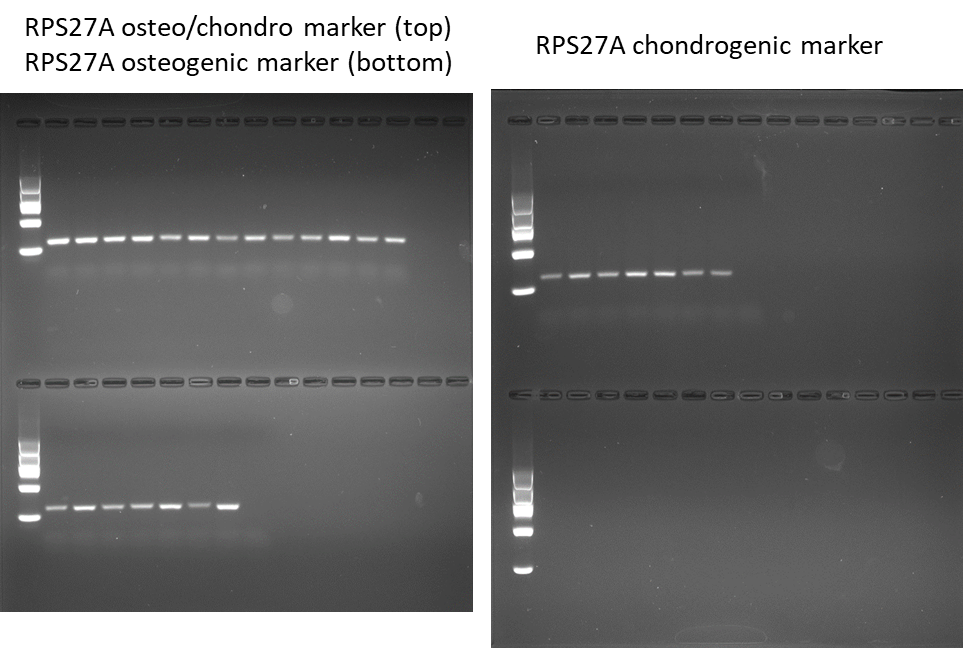


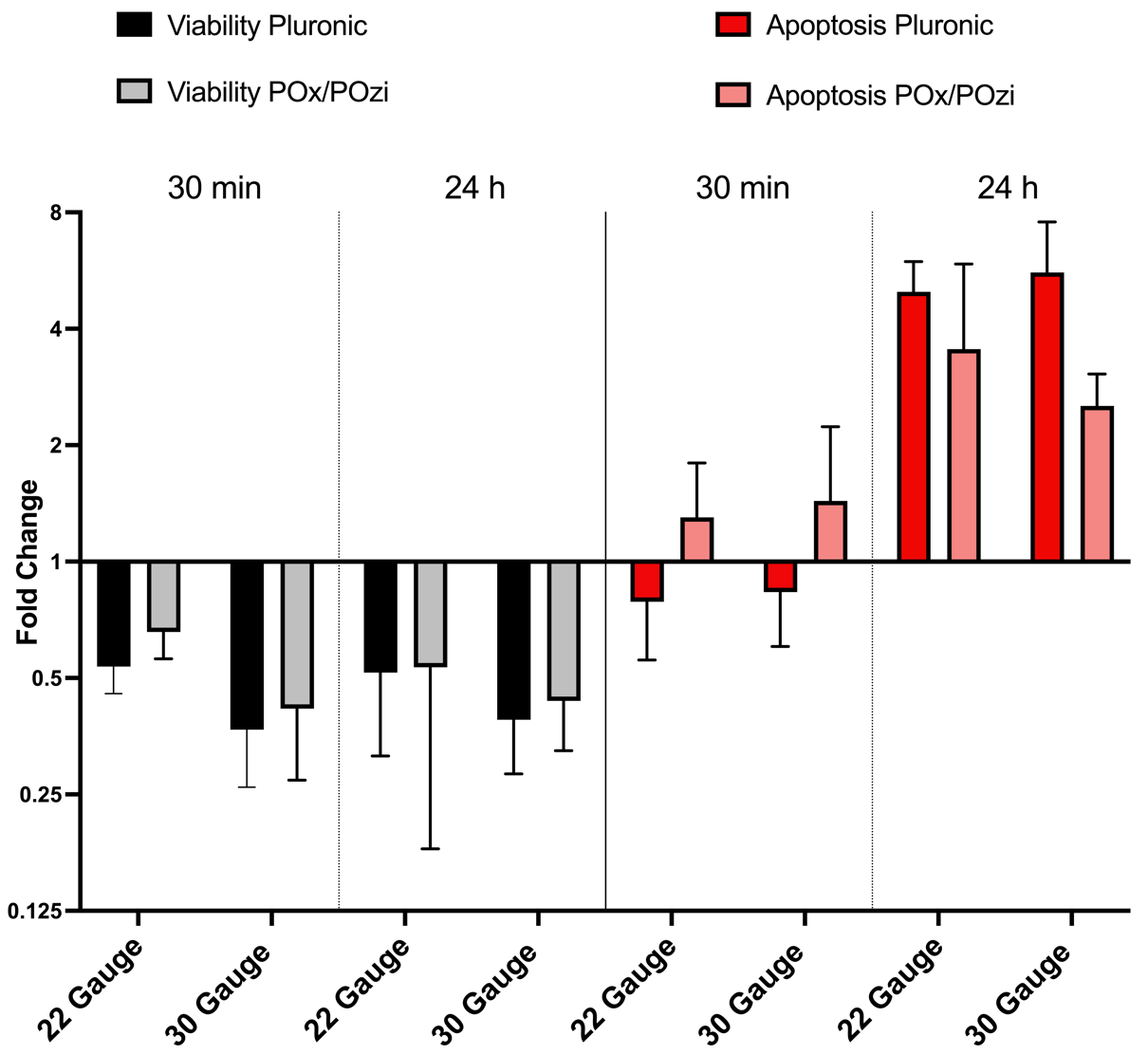


**Supplementary Figure 4.** Viability and apoptosis measurements of the printed bioink after 30 min and 24 h of incubation are depicted. Viability and apoptosis were assessed using Promega’s CellTiter-Glo® and Caspase-Glo® 3/7 assays, respectively, following the manufacturer’s protocols. 3000 cells were seeded per well in white, 96-well plates. The number of independent experiments for Pluronic is n = 3, and for POx/POzi, it is n = 4. The fold change expression is normalized to a split control measured individually daily. The mix control was not quantified, and comparisons were made only between the printed constructs at the time points showing the largest viability differences.
